# Attitude Corrections for Sounding Rocket Dropsondes Using Magnetometer and Langmuir Probe Measurements

**DOI:** 10.3390/s26185690

**Published:** 2026-09-08

**Authors:** Henry Valentine, Joshua Milford, Aroh Barjatya, Nathan Graves, Robert Clayton

**Affiliations:** Center for Space and Atmospheric Research, Embry-Riddle Aeronautical University, 1 Aerospace Blvd, Daytona Beach, FL 32114, USA; milfordj@my.erau.edu (J.M.); gravesn@erau.edu (N.G.); claytor8@erau.edu (R.C.)

**Keywords:** attitude correction, Langmuir probe, magnetometer

## Abstract

In this work, we present a method for correcting attitude-dependent fluctuations in fixed-bias Langmuir probe ion density measurements from small, spin-stabilized sounding rocket dropsondes. The approach resolves dropsonde attitude using three-axis magnetometer data, observations from the Langmuir probe itself, and an analytical model of the sonde’s rotational motion. Together, these inputs constrain the dropsonde’s angular momentum vector in inertial space and enable reconstruction of its time-resolved attitude without requiring prior knowledge of the sonde’s orientation at deployment. The derived attitude solution is then used to correct the Langmuir probe ion density measurements for orientation-dependent changes in collection geometry caused by the sonde’s precessional motion. This technique is applied to data from the August 2022 Sporadic E Electrodynamics Demonstration (SpEED Demon) sounding rocket campaign, in which four dropsondes were ejected. Validation is performed by comparing attitude-corrected dropsonde ion density measurements to those of the main payload in a near-coincident measurement regime. The corrected data show strong agreement with the main payload observations, demonstrating that this technique can recover physically consistent ion density profiles from spin-stabilized dropsonde measurements.

## 1. Introduction

During several recent NASA-funded sounding rocket campaigns, small form-factor ejectable dropsonde subpayloads have been deployed to collect spatially distributed in situ measurements of local electrodynamic and plasma quantities in Earth’s ionosphere [1,2,3,4,5]. These low Size, Weight, and Power (SWaP) devices are ejected from a main payload and serve to augment the capabilities of single-trajectory, instrumented sounding rocket platforms. The use of dropsondes enables simultaneous, multi-point measurements in the ionosphere without incurring significant additional financial or logistical strain on the host program [6]. One such campaign, the Sporadic E Electrodynamics Demonstration mission (SpEED Demon), launched from NASA’s Wallops Flight Facility on 24 August 2022 and deployed a suite of four ejectable dropsonde subpayloads to collect distributed ion density and magnetic field measurements [1]. The combined purpose of the instrumented main payload and subpayloads was to observe and characterize vertically narrow regions of enhanced plasma density in the lower ionosphere. These enhancements, known as Sporadic E, are common in the middle-latitude summer post-sunset ionosphere below 130 km and are generally associated with strong vertical shears in the neutral wind [7,8,9]. The use of dropsonde subpayloads on SpEED Demon provided a unique opportunity to resolve small-scale (<1 km) horizontal structures within one such layer during a single rocket flight [6].

A notable challenge encountered when analyzing data for this campaign was the sensitivity of dropsonde ion density measurements to each spacecraft’s attitude. The SpEED Demon dropsondes were spin stabilized and employed no active attitude control systems (ACS). Furthermore, data dropouts and an anomalous main payload ACS response during the dropsonde deployment prevented accurate initial estimates of subpayload orientation from being obtained, thus precluding the use of many standard attitude determination methods. For these reasons, novel and accurate attitude determination and correction techniques were required in order to accurately evaluate and compare observational data from the dropsondes.

For low-cost, ejectable sounding rocket dropsondes, attitude determination sensor availability is severely limited. Star trackers are too large and expensive for this application, and sun sensors are not applicable for nighttime launches, such as SpEED Demon. Classical attitude determination methods, such as the TRIAD algorithm, require two or more linearly independent reference vectors to fully constrain the payload orientation in an inertial reference frame [10]. More general nonlinear attitude estimation methods can combine gyroscope propagation with vector measurements [11]; however, these approaches require adequate initial attitude estimates, sufficiently stable gyroscope measurements, and enough measurement history for convergence. Similar approaches have been developed specifically for rapidly spinning vehicles, combining geomagnetic measurements with gyroscope measurements, rotational dynamics, or filtering techniques to recover otherwise underconstrained attitude states [12,13,14]. In the case of SpEED Demon, data dropouts, the main payload ACS anomaly, and inadequate gyroscope stability prevented reliable propagation from a known initial attitude.

The primary dropsonde attitude sensor was the PNI RM3100 three-axis magnetometer. This alone is insufficient for complete instantaneous attitude determination because a single magnetic-field vector constrains only two degrees of freedom, leaving rotation about the magnetic-field direction unresolved. Magnetometer-only attitude determination methods do exist [15,16], but these generally rely on measurements of the magnetic field vector and its derivatives over the course of one or more Earth orbits. Since SpEED Demon observed minimal variation in the magnetic field along its short suborbital trajectory, this technique was not viable for the presented case. Roberts et al. [17] approached this same problem for a similar set of dropsondes flown on an October 2015 sounding rocket test flight. The authors successfully developed an attitude solution using magnetometer data, but their method relied on accurate initial knowledge of dropsonde orientation in an Earth-fixed reference frame. Since this information was not available in the case of SpEED Demon, a modified approach is required.

In this article, we present a novel method of attitude determination and measurement correction for the SpEED Demon dropsondes that does not require specific knowledge of the initial payload orientation. Using three-axis magnetometer data and Langmuir probe current measurement variations that correspond to the ion ram direction, an accurate solution is obtained. The approach is validated by comparing the attitude-corrected plasma density measurements for each SpEED Demon dropsonde to the known absolute density acquired from the main payload during the upleg sporadic E altitude regime where all payloads provided near-coincident measurements.

## 2. The SpEED Demon Dropsondes

**Figure 1:**
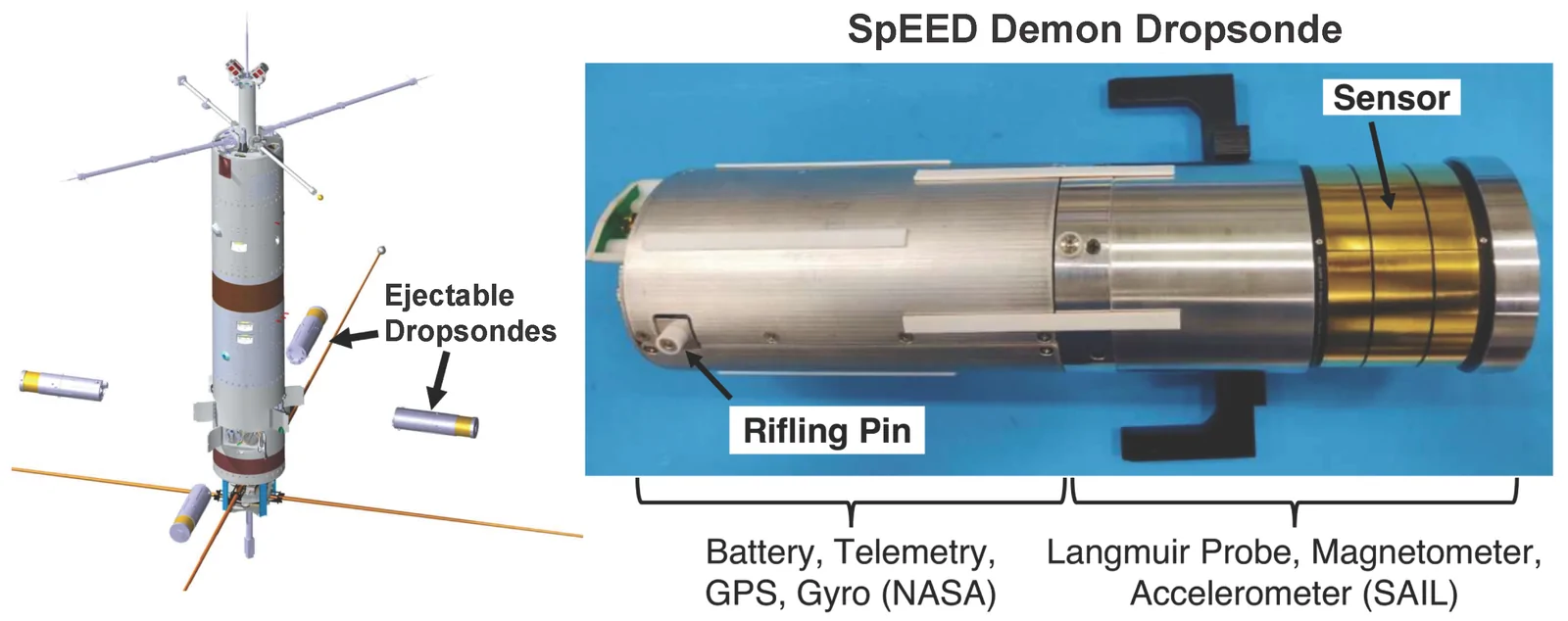
(**Left**) The SpEED Demon sounding rocket main payload with four ejectable dropsondes being released. (**Right**) One of the SpEED Demon dropsondes. The dropsonde dimensions are 33.5 cm (length) by 8.6 cm (diameter). The gold-plated Langmuir probe sensor collects ion current from the plasma. The black structure is a stand used to protect the sensor during integration.

**Figure 2:**
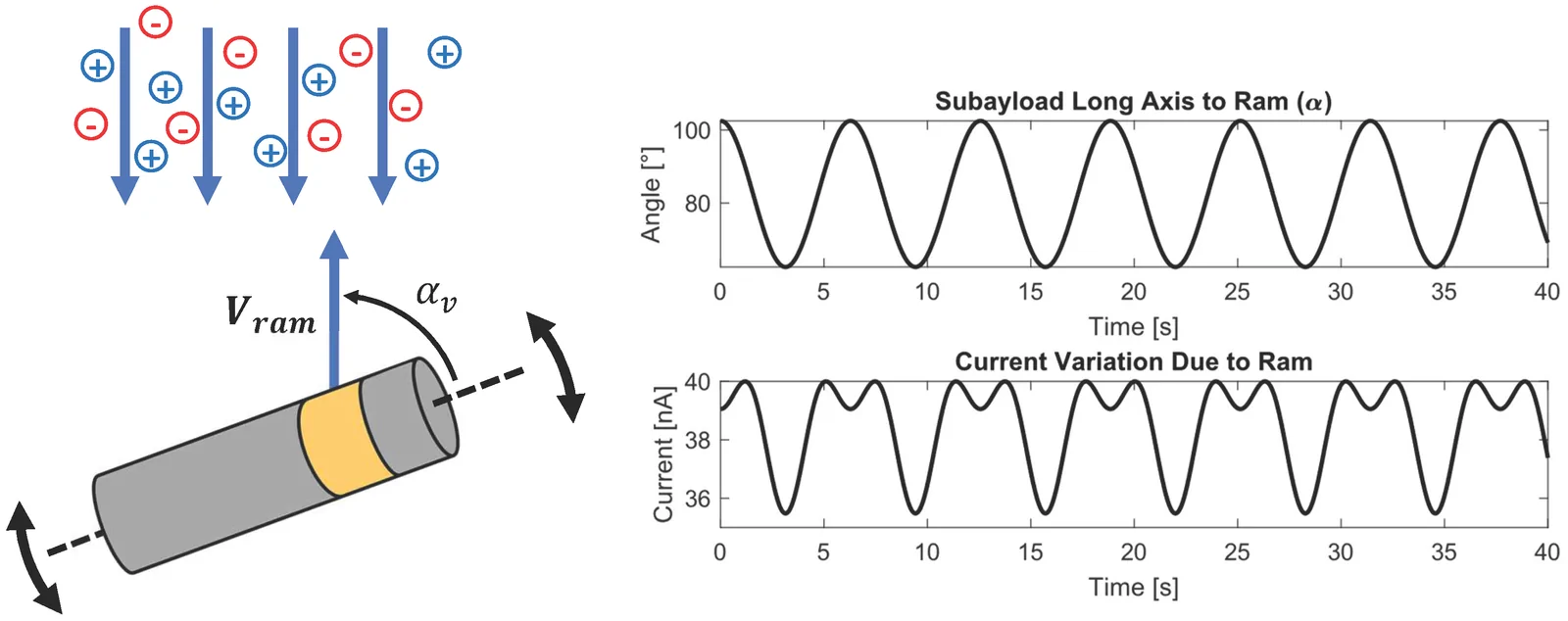
Variation in collected current for the Positive Ion Probes (PIPs) caused by the precession or “coning” motion of the dropsondes. The particular shape of this variation (lower plot) can be used to constrain the dropsonde attitude solutions given a model of the system’s motion and accurate knowledge of the ram direction from Global Positioning System (GPS) velocity measurements.

Figure 1 depicts the main rocket payload and one of four ejectable dropsondes flown on the SpEED Demon sounding rocket mission. Each dropsonde was composed of an instrumentation segment and a NASA-provided telemetry and power segment. All hardware was contained within a 33.5 cm (length) by 8.6 cm (diameter) cylindrical housing, and rifling pins were used to spin-stabilize each dropsonde about its long axis on deployment. The dropsondes were each stored in a spring-loaded tube on the main payload and ejected 60 s after liftoff with a horizontal velocity of approximately 4 m/s. In addition to power and telemetry, the NASA segment included a 9 degree-of-freedom inertial measurement unit (LSM9DS1), which contained a gyroscope, magnetometer, and accelerometer. The primary instrumentation suite consisted of a PNI RM3100 three-axis magnetometer, an Analog Devices ADXL355 three-axis accelerometer, and a Positive Ion Probe (PIP) developed by the Space and Atmospheric Instrumentation Lab. The PIP is a fixed-bias Langmuir probe designed to measure relative changes in ion density throughout the flight (see [6,18,19]). The sensor (shown in Figure 1) is a gold-plated flexible printed circuit board (PCB) that wraps around the payload cylinder and collects an electric current from the plasma, which—in a first-order approximation—is proportional to the local ion density. For large, ion-collecting sensors such as the ones installed on the dropsondes, a substantial—or in many cases, dominant—source of current to the instrument is produced by the ram velocity of the plasma in the sensor-fixed reference frame. This is primarily a consequence of the payload’s velocity through the plasma (>1 km/s for sounding rocket flights), which significantly exceeds the thermal velocity of ions in the local environment (typically < 400 m/s for altitudes below 120 km). The ram component of the collected current $I_{ram}$ varies according to the orientation of the sensor and is given by the following expression:

(1) $$I_{ram}=q_{i}n_{i}v_{ram}A_{cross}\sin\alpha_{v}$$

where $q_{i}$ is the ion species charge, $n_{i}$ is the ion number density, $v_{ram}$ is the ion ram velocity, $A_{cross}$ is the cross-sectional area of the sensor as viewed from a radial axis of the dropsonde cylinder, and $\alpha_{v}$ is the angle between the long axis of the payload and the ram direction as depicted in Figure 2. It is more traditional to express Equation (1) in terms of the cosine of the angle between the ram direction and the sensor surface normal [18]; but given the axial symmetry of this probe, the complementary angle $\alpha_{v}$ is more convenient since the collected current is invariant under a rotation about the dropsonde’s long axis. The ram velocity vector, $v_{\mathrm{ram}}$, is taken to be the payload velocity as determined by Global Positioning System (GPS) measurements.

The SpEED Demon attitude control system was supposed to arrest the angular velocity of the spin-stabilized main payload prior to horizontally ejecting the dropsondes; however, a staging anomaly resulted in deviations from the expected mass distribution. Although the payload was able to orient itself vertically, the ACS did not completely stop its rotation and the residual motion coupled with frictional forces within the deployment tubes imposed momentary, lateral torques on the dropsondes during ejection. These torques induced a precession—or “coning” motion—for each dropsonde that persisted throughout the flight trajectory. This motion led to periodic fluctuations in the measured PIP signal. Per Equation (1), the collected current depends on the foreshortened area of the sensor that is exposed to the ram direction. Thus, as the dropsonde precesses, the resulting shape of the current variation in an otherwise smooth plasma density environment is uniquely determined by the angle between the ram direction and the cylindrical body, as shown in Figure 2. Given that the vertical component of the dropsonde velocity is greatest at low altitudes, the dropsondes were ejected horizontally in order to maximize sensor exposure to ram during critical measurement regimes. However, precessional motions combined with slow changes in the ram direction due to the parabolic trajectory of the dropsondes caused measurement variations which need to be accounted for when interpreting probe data to resolve local ion densities. Therefore, an accurate attitude determination and correction method is required.

## 3. Dropsonde Attitude Determination

### 3.1. Magnetometer Calibration and Alignment to Spin Axis

**Figure 3:**
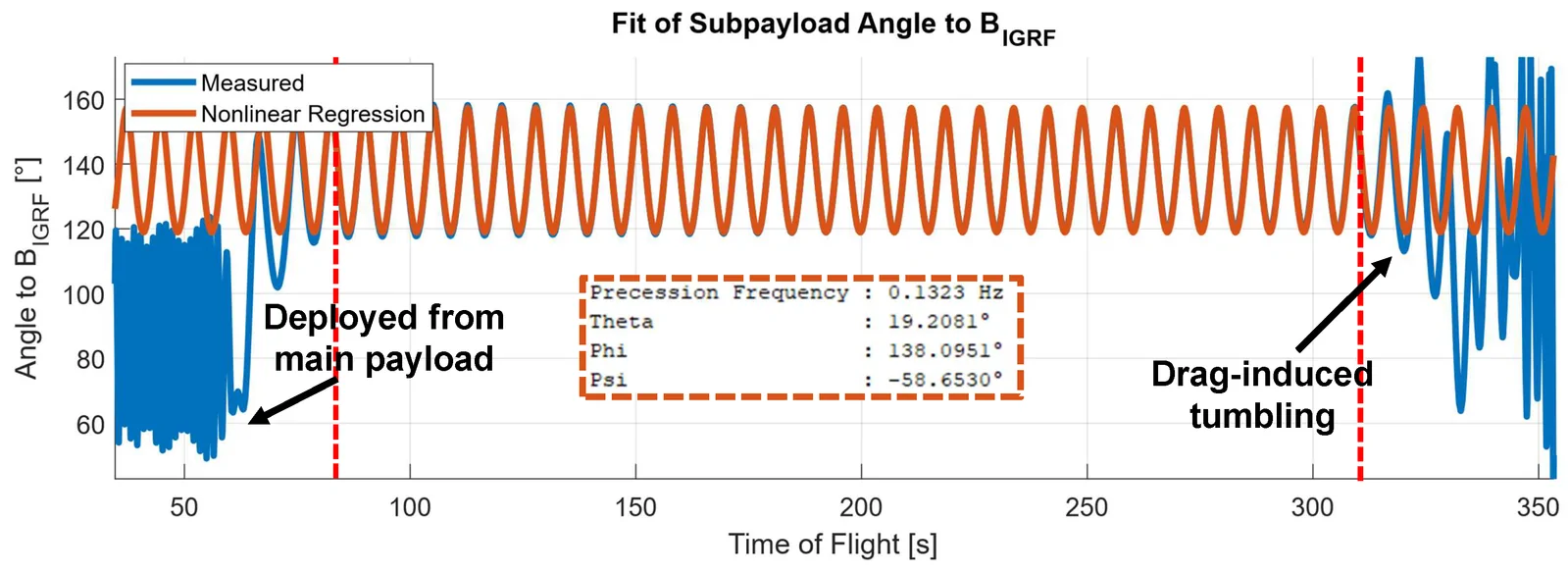
A nonlinear regression fit applied to the measured angle between the dropsonde spin axis and the magnetic field direction $\alpha_{B}$ for dropsonde 1. The regression model is described in Equation (4) and the fit yields values for $\theta_{y}$, $\phi_{B}$, $\psi_{B}$, and the precession frequency $\omega_{p}$ as depicted in Figure 4. Here, these values are expressed in degrees and Hz rather than radians and rad/s for the purpose of readability. Dropsonde ejection occurs at approximately 75 km on the upleg and drag forces begin to induce tumbling below 85 km on the downleg. The dashed red lines indicate the times at which the dropsondes passed through the observed sporadic E layer on the upleg and downleg of the flight.

In solving for the dropsonde attitudes in an Earth-fixed reference frame, we begin by performing an in-flight magnetometer calibration and alignment for each dropsonde. This process is described thoroughly by Roberts et al. [17], so we provide only a cursory treatment here and refer the interested reader to their paper for further details. The magnetometer calibration is accomplished by scaling and offsetting the observed magnetic field measurements to match the output of an International Geomagnetic Reference Field (IGRF) model run [20] obtained for the full, time-stamped trajectories of the SpEED Demon dropsondes. We then apply a transformation to these magnetometer measurements that improves alignment between the *y* component of the magnetic field data and the long axis of the dropsonde. This corrects for any small alignment errors in the mounting orientation of the magnetometer. The calibrated and aligned magnetic field data can then be used to provide an accurate measure of the angle between the dropsonde’s long axis, henceforth referred to as the *y*-axis, and the magnetic field direction (shown for dropsonde 1 in Figure 3). Knowledge of this time-varying angle, in addition to the IGRF magnetic field vector in the local east-north-up coordinate frame, yields an attitude solution for each dropsonde up to a rotation about the magnetic field vector. In other words, the angle between the dropsonde and the magnetic field direction is known at all times during flight, but the attitude is not fully constrained because the magnetometer measurement is invariant under a rotation of the dropsonde about an axis that aligns with the magnetic field.

**Figure 4:**
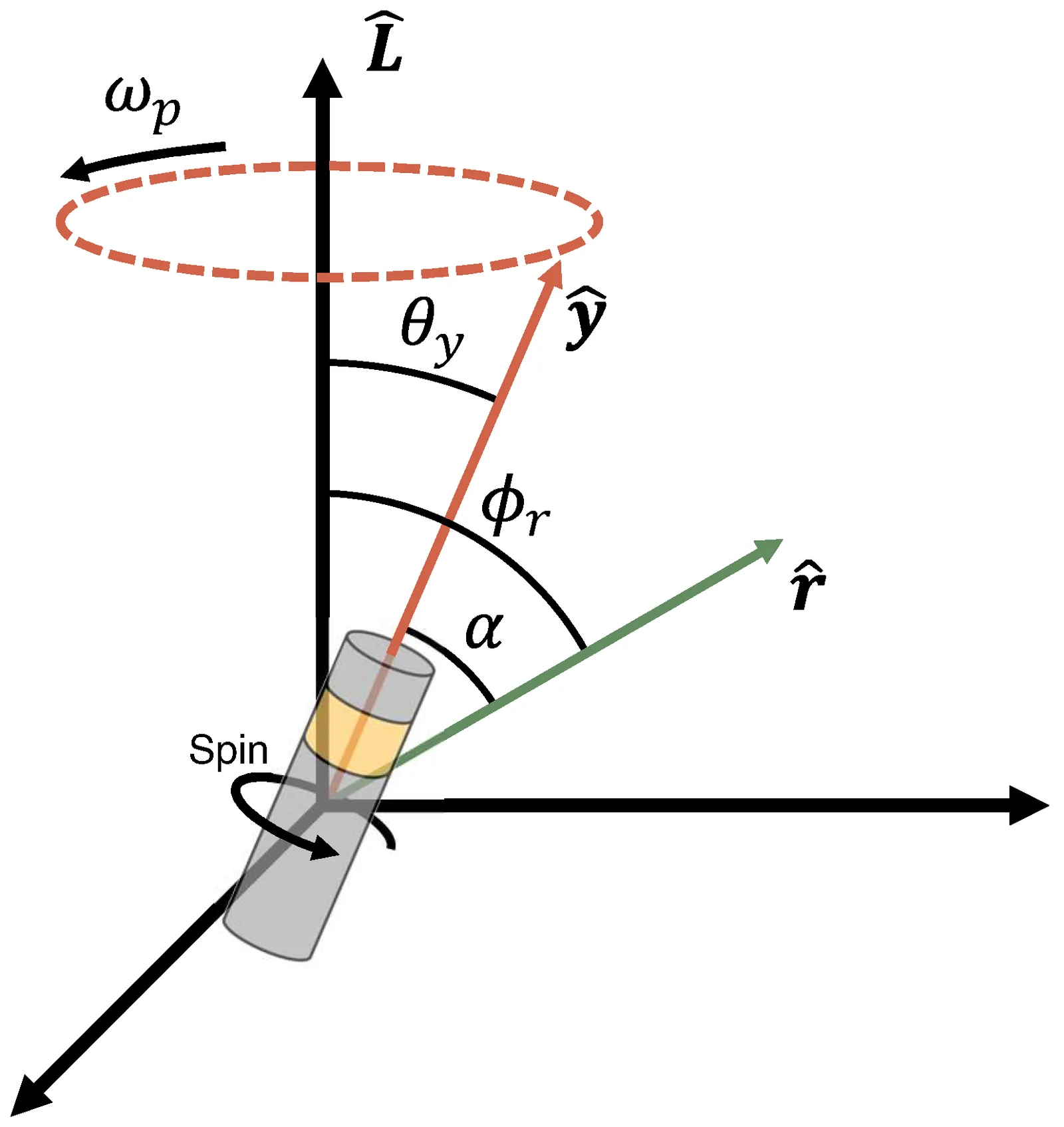
Rotational motion of a SpEED Demon dropsonde in an inertial reference frame. The constant angular momentum vector $\hat{L}$ is chosen as an axis for convenience. Angular relationships between the dropsonde’s long axis $\hat{y}$, $\hat{L}$, and an arbitrary vector $\hat{r}$ are depicted. $\hat{r}$ is taken to represent the magnetic field direction or the payload velocity direction during different stages of this analysis.

### 3.2. Modeling the System

To fully constrain the attitude of each dropsonde, we present an analytical model of its rotational motion and relate this to the magnetic field and ram directions in the Earth-fixed frame. Although the dropsonde inertia matrices were not directly computed or measured, the mass distribution was mostly symmetric about the *y*-axis, so it is reasonable to approximate the equations of motion as those of a prolate, cylindrical rigid body under torque-free rotation [21]:

(2) $$I\dot{\omega }+\omega \times \left(I\omega \right)=0$$

where ω is the angular velocity of the cylinder in the body-frame and the moment of inertia tensor is given by

(3) $$I=\left[\begin{array}{ccc} I_{T} & 0 & 0\\ 0 & I_{y} & 0\\ 0 & 0 & I_{T} \end{array}\right]$$

where $I_{y}$ is the moment of inertia about the long axis of the dropsonde and $I_{T}$ is the moment of inertia about any radial axis. For a prolate cylinder, $I_{T}>I_{y}$. The solution to Equation (2) is represented graphically in Figure 4. Following its ejection from the main payload, each dropsonde cylinder is therefore assumed to undergo constant-rate rotation about its long axis $\hat{y}$ and constant-rate precession about its angular momentum vector L. Aerodynamic torques remain negligible above ∼90 km, so the angular momentum vector is assumed to be fixed in inertial space. The vertical axis of our chosen reference frame is thus assigned to coincide with the unit vector $\hat{L}$ as depicted in order to simplify calculations. Obtaining the direction of $\hat{L}$ in addition to the amplitude $\theta_{y}$ and phase of the dropsonde precessional motion is sufficient to determine its orientation in inertial space for all timestamps and provide the necessary attitude corrections to accurately interpret ion density measurements.

### 3.3. Constraining $\hat{L}$ with Magnetometer Measurements

Combining this model with magnetometer measurements for a given dropsonde allows us to constrain the possible solution space for $\hat{L}$. Figure 4 depicts the angular relationships between the dropsonde spin axis $\hat{y}$, the angular momentum vector $\hat{L}$, and an additional unit vector $\hat{r}$, which we will first take to represent the magnetic field vector. Using this diagram, we geometrically derive the following expression that describes the angle, α, between the spin axis and $\hat{r}$:

(4) $$\cos\alpha =\sin\phi \sin\theta_{y}\sin\left(\omega_{p}t+\psi \right)+\cos\phi \cos\theta_{y}$$

where $\omega_{p}$ is the precession rate of the spin axis, *t* is the time of flight, and ψ is the precession phase argument. $\theta_{y}$ and ϕ are the precession amplitude and angular difference between $\hat{L}$ and $\hat{r}$ as depicted. In the case that $\hat{r}$ represents the magnetic field direction, we will use the subscript “_*B*_” for all angles associated with this vector. (Later in our analysis, $\hat{r}$ will be used to represent the payload velocity direction and we will use the subscript “_*v*_”). Applying a nonlinear regression in the form of Equation (4) to the measured angle between the dropsonde spin axis and the magnetic field yields solutions for the values of $\omega_{p}$, $\theta_{y}$, $\psi_{B}$, and—most notably—$\phi_{B}$. The regression performed on data from dropsonde 1 is shown in Figure 3. As stated, $\hat{r}$ represents the magnetic field direction in this case, so $\phi_{B}$ provides an angle between $\hat{L}$ and the known magnetic field vector, which is essentially constant throughout the flight. Thus, the direction of $\hat{L}$ is constrained to a cone of possible vectors that form a constant angle with the external magnetic field, B, and we are able to parameterize it as follows:

(5) $$\hat{L}=\hat{B}\cos\phi_{B}+\left({\hat{T}}_{1}\cos\tau +{\hat{T}}_{2}\sin\tau \right)\sin\phi_{B}$$

where τ is a free parameter, and the unit vectors ${\hat{T}}_{1}$ and ${\hat{T}}_{2}$ represent any arbitrarily selected pair of orthogonal basis vectors that lie within the plane perpendicular to $\hat{B}$. Visually, the term $\left({\hat{T}}_{1}\cos\tau +{\hat{T}}_{2}\sin\tau \right)$ traces out a circular path around the magnetic field vector.

### 3.4. Constraining $\hat{L}$ with Instrument Measurements

To this point, $\hat{L}$ has been determined to within a single degree of freedom, but more information is required to fully specify its direction. In the work by Roberts et al. [17], the initial orientation of the dropsonde is obtained through a relative transformation of the main payload’s attitude at the time of ejection. In the case of SpEED Demon, this technique is not reliable due to the aforementioned ACS issues and data dropouts experienced during flight. Instead, we specify the final direction of $\hat{L}$ by considering the particular shape of measured ram current variations in the PIP data.

**Figure 5:**
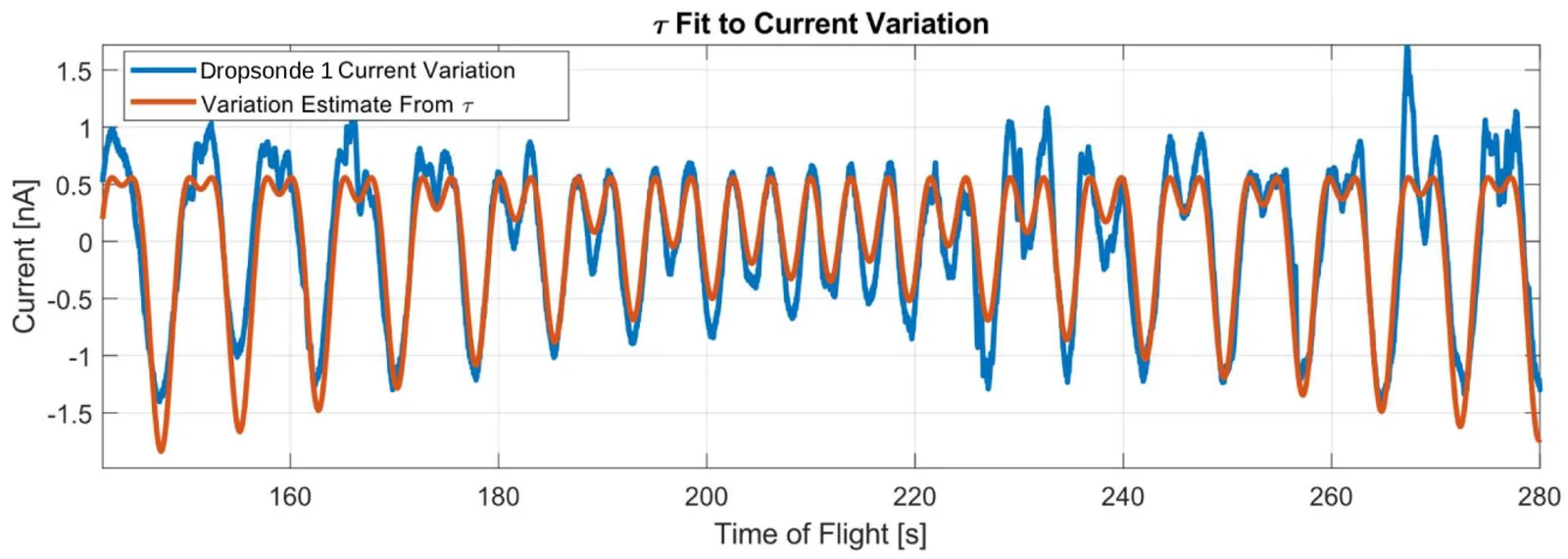
(Blue curve) Measured PIP current data which have been detrended to remove any dependence on dropsonde velocity magnitude and plasma density. Data shown are for the smoothly varying density region above ∼130 km. (Orange curve) The results of a nonlinear optimization algorithm that varies parameters within our model of dropsonde rotation and current collection to best fit the measured data. The optimization yields a value for the free parameter τ which fully specifies the dropsonde angular momentum vector according to Equation (5).

Figure 5 (blue curve) shows the attitude-induced current fluctuations observed by dropsonde 1 in the otherwise smooth density region above ∼130 km. A detrending operation was applied to remove any long-scale measurement variations caused by changes in ion density, spacecraft charging or dropsonde velocity magnitude. The shape of this curve directly relates to the time-varying angle between the ram vector and the dropsonde *y*-axis as specified by Equation (1) and depicted in Figure 2. Therefore, in order to fully constrain $\hat{L}$, we perform a nonlinear optimization to obtain a value of the free parameter τ that minimizes the difference between the observed current variation and that which is predicted by our attitude solution.

The optimization problem details are as follows: Selecting a value of τ between 0 and 2π specifies the vector $\hat{L}$ according to Equation (5). The angle $\phi_{v}$ between $\hat{L}$ and the dropsonde GPS velocity direction $\hat{v}$ is then computed from the dot product of those two vectors. The angle $\alpha_{v}$ between $\hat{v}$ and $\hat{y}$ is determined using Equation (4) where $\phi =\phi_{v}$, $\theta_{y}$ and $\omega_{p}$ are reused from the previous fit of the magnetometer data (see Figure 3), and $\psi_{v}$ is allowed to vary as a fit parameter to ensure phase alignment with the PIP measurements. The estimated current fluctuations $I_{est}$ over the selected interval (the orange curve in Figure 5) are then obtained by

(6) $$I_{est}=C_{1}\sin\alpha_{v}+C_{0}$$

where the constant coefficients $C_{1}$ and $C_{0}$ (with units of nA) are optimization variables, and a velocity term is unnecessary because the measured data have been detrended. The final optimization algorithm seeks to minimize the difference between the measured current fluctuations (blue curve in Figure 5) and the estimated current fluctuations (from Equation (6)) by varying τ, $\psi_{v}$, $C_{1}$, and $C_{0}$. The resulting value of τ specifies a direction for the angular momentum vector that is physically consistent with the measured current data. $\hat{L}$ combines with the previously acquired values of $\omega_{p}$, $\theta_{y}$, and $\psi_{B}$ from the magnetometer data to fully specify $\hat{y}$ as a function of time in the Earth-fixed frame. Hence, the dropsonde attitude is determined to within a rotation about its long axis. The remaining degree of freedom could easily be constrained using magnetometer data; nevertheless, we neglect to do so as the rotation of the dropsonde about its *y*-axis has no effect on the measured current and is therefore not relevant to the present analysis.

## 4. Attitude Correction and Validation

To validate the acquired attitude solution and obtain accurate measurement corrections, we consider the dropsonde current observations shortly after deployment. At ∼102 km altitude (83 s flight time), all dropsondes were within 70 m of the main payload and flew through a sporadic E plasma density enhancement. On account of their relative proximity, it is reasonable to assume that the density profile measured by each dropsonde should be roughly consistent with that of the main payload in this region. The SpEED Demon main payload instrumentation provides accurate in situ observations of ion and electron densities and is not subject to the substantial attitude-induced modulations seen in the dropsonde data. We therefore take the main payload measurements to be the “ground truth” in our analysis and assume that all dropsonde density measurements should approximately match this profile shortly after ejection. If this is shown to be the case after attitude-dependent corrections are applied to the dropsonde current measurements, the derived attitude solutions and attitude correction technique will have been validated.

**Figure 6:**
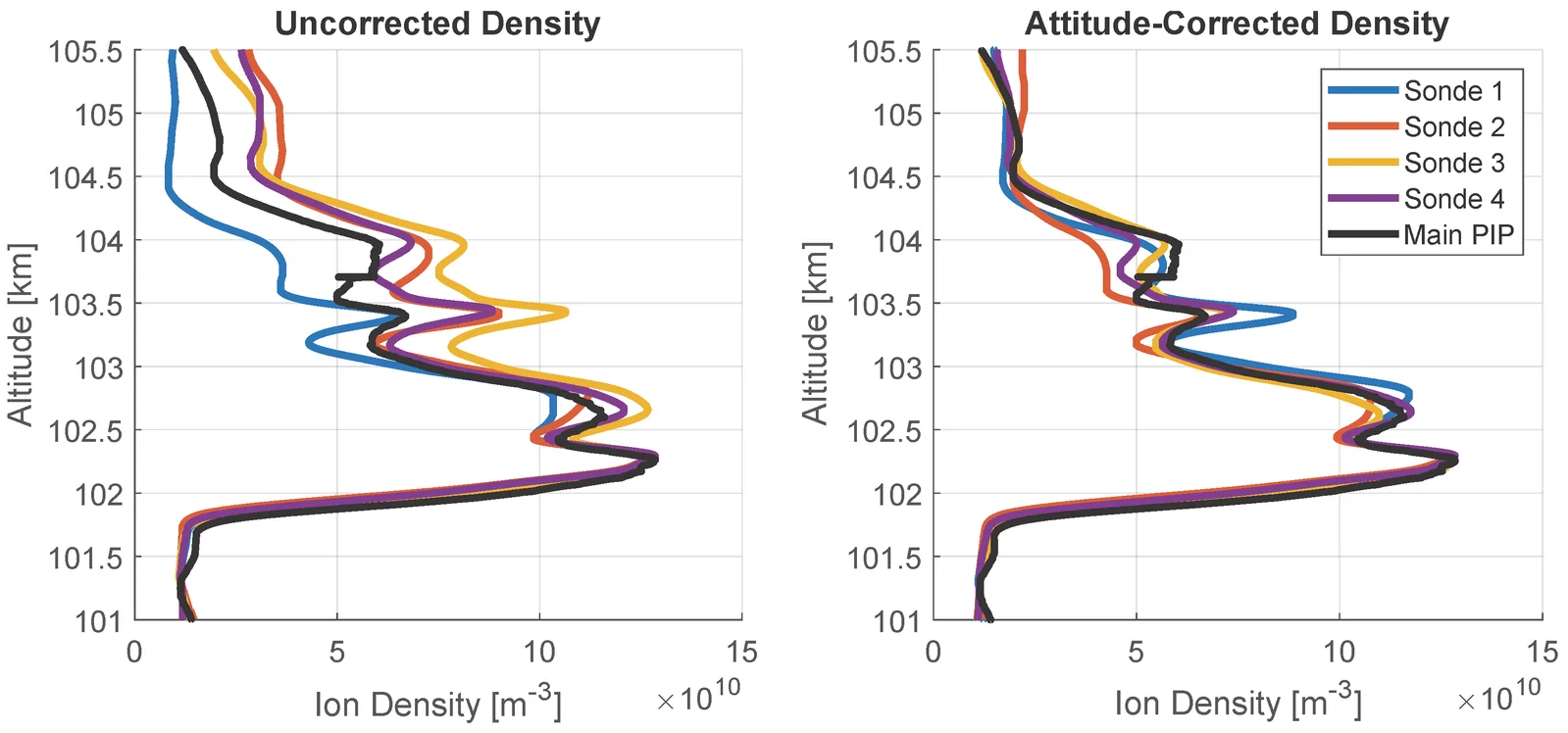
SpEED Demon dropsonde and main payload ion density measurements in an enhanced plasma layer shown before (**left**) and after (**right**) attitude corrections were applied. A discontinuity, appearing in the main payload data just below 104 km is associated with a staging anomaly and is non-physical. The dropsondes were within 70 m of the main payload at this time, so the “collapsing” of the dropsonde density measurements to match those of the main payload validate the accuracy of our attitude solutions.

The dropsonde PIP instrument provides only relative ion density measurements. For this reason, the measured currents must be normalized and scaled to match an absolute density reference from the SpEED Demon main payload. This is shown in the left panel of Figure 6 where the data from each dropsonde were scaled to match the main payload density before entering the sporadic E layer at 101.2 km and upon reaching the layer peak at 102.2 km. The resulting profiles clearly show the effect of attitude-induced modulation in this critical measurement regime as the measurements quickly diverge above the selected scaling points.

To account for attitude-driven ram current variations in the dropsonde PIP data, a time-dependent additive correction factor $I_{correction}$ is obtained by differencing Equation (1) with itself for the cases of *maximum* possible ram current ($\alpha_{v}=90^{\circ }$) and expected ram current (computed using our attitude solution). Performing this operation, we have the following:

(7) $$I_{correction}=\left(I_{\alpha =90^{\circ }}-I\right)=-q_{i}n_{i}A_{cross}v_{ram}\left(t\right)\left(1-\sin\alpha_{v}\left(t\right)\right)$$

When added to the measured PIP data, this correction factor yields the current that *would* be observed if the PIP sensor were perfectly ram-facing throughout the entirety of the flight; thus, removing the attitude-dependent current modulations. The dependence of Equation (7) on ion density presents a challenge given the fact that $n_{i}$ is the target observable of this instrument and is therefore unknown ahead of time. Again, we assume that the measured current and ion density are directly proportional in a first-order approximation, so we replace $n_{i}$ with the measured current *I* in Equation (7) and drop the constant terms in favor of the following proportionality:

(8) $$I_{correction}=\left(I_{\alpha =90^{\circ }}-I\right)=C_{2}I\left(t\right)v_{ram}\left(t\right)\left(1-\sin\alpha_{v}\left(t\right)\right)$$

In applying the additive correction term described in Equation (8), the linear scale factor $C_{2}$ (units of s/m) is computed for each dropsonde, which results in density profiles that best fit the main payload density data shortly after ejection. Figure 6 shows the densities for all four dropsondes and the main payload, before and after attitude corrections were applied within the enhanced-density sporadic E layer encountered on the upleg of the flight. The resulting profiles show excellent agreement with the main payload in a near-coincident altitude regime, providing validation of the attitude estimation process that was developed using data from 140–280 s flight time, but applied to the enhanced density layer between 83 and 87 s. Equation (8) was further applied to evaluate correction currents throughout the flight of each sonde using the coefficients evaluated for the upleg regime.

## 5. Discussion

### 5.1. Discussion of Uncertainty

Given the multi-stage fitting methodology required to obtain the dropsonde attitude corrections, we choose to evaluate and bound the uncertainties associated with the corrected measurements through a comparison with the presented validation case. Prior to correction, the dropsonde profiles exhibit clear attitude-dependent deviations from the main payload absolute density profile, as shown in the left panel of Figure 6. The precession frequency of each dropsonde was approximately 0.1 Hz, corresponding to a vertical spatial period of roughly 8 km. Over the altitude range depicted in Figure 6, the measurements include approximately one half rotation of each dropsonde about its angular momentum vector, and the largest relative errors in the uncorrected density data are expected just above 105 km, one half of a spatial period above the sporadic E peak where all dropsonde measurements were scaled to match the main payload.

The percent root mean squared error (RMSE) between each dropsonde profile and the main payload profile was computed before and after applying attitude corrections. These values are summarized in Table 1. The uncorrected upleg profiles had percent RMSE values ranging from 11.4% to 15.8%. After correction, the corresponding RMSE values were reduced to 3.9–7.5%, and the density profiles visually collapse to the main payload in Figure 6, indicating that the attitude-dependent modulation was substantially reduced.

The residual differences between the corrected dropsonde profiles and the main payload should not be strictly interpreted as errors in the attitude solution. Although the payloads were nearly coincident within the upleg sporadic E layer, they did not sample identical plasma volumes. Some spatial variability in ion density is expected within the validation region given the dynamic and potentially turbulent nature of low-altitude sporadic E layers [22]. Consequently, some disagreement between the individual dropsonde and main payload profiles is expected even for a perfect attitude correction. The corrected density profiles should align closely given the proximity of observations, but this spatial variability may contribute to the differences in corrected RMSE observed among the four dropsondes.

Additional sources of uncertainty in the obtained attitude corrections arise from variations in the PIP current that are unrelated to payload attitude. The attitude-fitting procedure was intentionally performed in the relatively smooth plasma-density region above the sporadic E layers, where long-timescale variations in density and spacecraft charging were slow, smooth, and relatively small. The measured current was detrended before fitting the attitude-dependent modulation, reducing sensitivity to slowly varying contributions from plasma density, bulk ion flow, spacecraft charging, and payload velocity magnitude. These effects are therefore unlikely to have strongly influenced the recovered attitude solution for the present flight.

Moderate dropsonde charging was observed in the measured PIP data over the course of the flight, and variations in spacecraft potential can alter the relationship between collected current and the local plasma density. The identified charging was slow compared to the dropsonde precession period and was therefore largely removed during the detrending procedure between the sporadic E layers; however, residual charging effects within the sporadic E layer may contribute to the differences between the corrected dropsonde and main payload density measurements. A more complete treatment of spacecraft charging could further improve the accuracy of future implementations of this correction technique.

Unmeasured bulk ion motion represents another potential source of uncertainty. The present analysis approximates the ion ram direction using the GPS-derived payload velocity. Any appreciable bulk ion flow would modify both the magnitude and direction of the relative ion ram velocity encountered by the probe and could therefore introduce current variations not represented by the attitude model. No independent measurements of the local ion or neutral velocity were available for the SpEED Demon dropsondes, preventing this contribution from being quantified directly. As with density and charging variations, however, variations in bulk ion velocity are expected to be slow in the fitted region above the sporadic E layer and would be mostly removed by the detrending operation. Nevertheless, this remains a potential source of systematic uncertainty.

Finally, the quantitative validation presented here is restricted to a relatively narrow altitude interval on the upleg. This region was selected because the dropsondes remained sufficiently close to the main payload that their measurements could reasonably be compared with a common absolute density reference from the main payload. No equivalent reference measurement was available during the remainder of the trajectory and this presents a notable limitation of the analysis and validation. The RMSE values presented in Table 1 should therefore be interpreted as a validation of the correction technique within this specific measurement regime rather than as a complete characterization of its accuracy over the entire flight.

Despite these uncertainties, the upleg comparison provides a useful bound on the magnitude of the attitude-dependent measurement error. The total disagreement between the uncorrected dropsonde and main payload profiles was less than approximately 16% in RMS and was reduced to 8% or less after correction. Because the remaining discrepancy also includes true spatial plasma variability and other instrumental effects, these values likely represent a conservative estimate of the residual uncertainty that may be attributed to the attitude correction itself.

### 5.2. Assessment of Model Assumptions and Solution Stability

The rotational model developed in Section 3 assumes that external aerodynamic torques are negligible at all scientifically relevant altitudes (i.e., in and above the sporadic E layer) and that the dropsonde mass distribution is approximately symmetric about its long axis. Under these assumptions, the dropsonde motion can be represented as that of an axially symmetric, prolate rigid body under torque-free rotation, yielding the analytical relationship provided in Equation (4).

Crucially, the moments $I_{T}$ and $I_{y}$ are never estimated explicitly. Instead, the precession frequency, amplitude, and phase are obtained by a regression of the magnetometer measurements throughout the flight. As a result, moderate departures from perfect axial symmetry may be accommodated by the fitted parameters provided that the resulting motion remains well represented by the functional form of Equation (4). Deviations from the assumed mass symmetry therefore influence the recovered attitude solution only to the extent that they cause the true rotational motion to depart from the modeled precession.

The validity of the stated approximations can be evaluated directly using the magnetometer measurements. The angle $\alpha_{B}$ between the dropsonde long axis and the magnetic-field direction is independently measured throughout the flight using the three-axis magnetometer and is also predicted by the rotational model through Equation (4). The difference between these two quantities provides an empirical measure of the degree to which the actual dropsonde motion departs from the assumed model, including the combined effects of imperfect axial symmetry and external torques.

**Figure 7:**
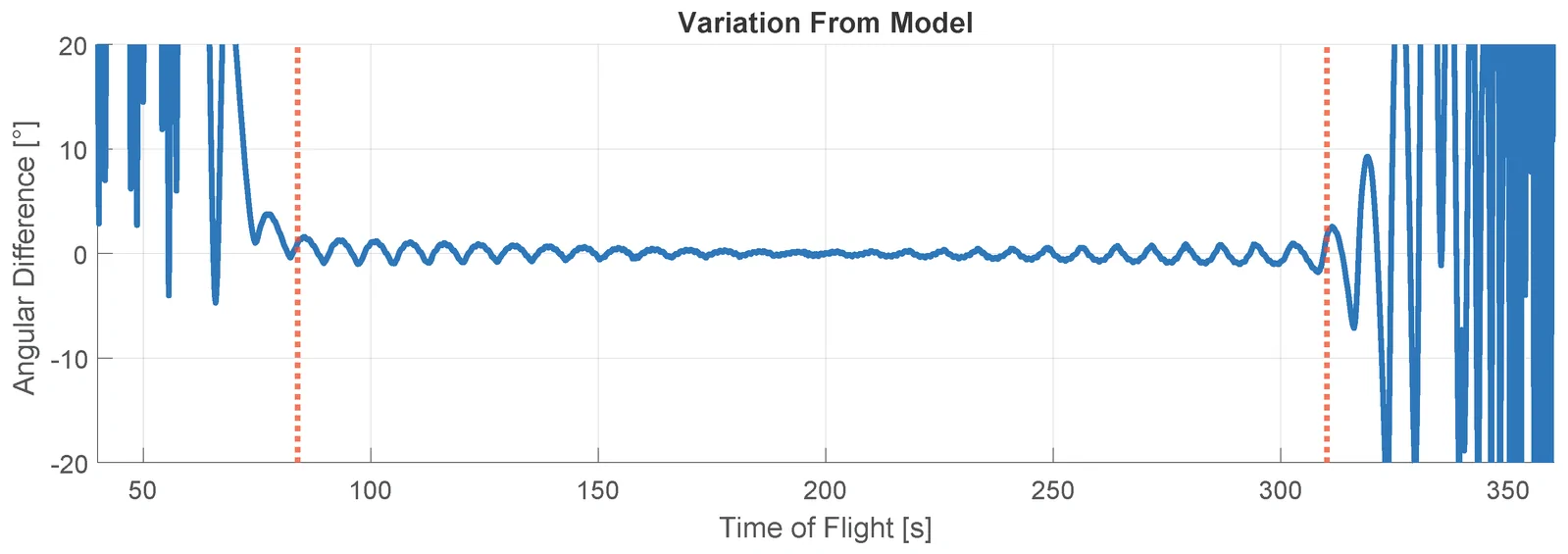
Difference between the measured and modeled angle $\alpha_{B}$ between the dropsonde long axis and the magnetic-field direction. The measured angle is obtained from the PNI three-axis magnetometer measurements, while the modeled angle is calculated using the nonlinear regression of Equation (4). The model residuals remain within 3° for all points between and including the sporadic E layer crossings (indicated by the vertical red dashed lines). This indicates that departures from the assumed torque-free, radially symmetric rotational model remain small during the flight regime that is relevant to scientific analysis. At lower downleg altitudes, increasing aerodynamic torques produce large departures from the model.

Figure 7 shows the difference between the measured and modeled values of $\alpha_{B}$ over the trajectory. The smallest residuals occur near the apogee, where atmospheric density and corresponding aerodynamic torques are smallest. The residuals gradually increase toward the lower-altitude portions of the trajectory, consistent with the increasing influence of aerodynamic forces. Nevertheless, between the upleg and downleg sporadic E measurement regions, indicated by the red dashed lines, the difference between the measured and modeled angles remains within a maximum of 3°.

Because the same rotational model is used to determine both the angle to the magnetic-field direction and the angle to the ion ram direction, the close agreement shown in Figure 7 provides confidence that the torque-free, approximately axisymmetric model adequately represents the dropsonde dynamics over the portion of the trajectory relevant to the present analysis. This comparison does not independently separate the contributions of aerodynamic torque, mass asymmetry, or other disturbances; however, it does bound their combined influence on the modeled rotational motion.

The sporadic E measurement regions occur near the limits of the trajectory over which the torque-free approximation remains most accurate. Although the model residual remains below approximately 3° in these regions, small accumulated differences in precession phase are present. Because accurate attitude determination was particularly important within the sporadic E layers, the modeled phase was locally adjusted to align with the measured variation in the relatively smooth plasma region immediately adjacent to each layer. This adjustment compensates for the accumulated phase error while retaining the precession amplitude and other parameters obtained from the fit.

### 5.3. Limitations and Further Applicability

A limitation of the presented attitude correction technique is that an external density reference is required to obtain accurately scaled ion density measurements. However, because the fixed-bias PIP does not independently provide an absolute ion density measurement, some form of external calibration is required regardless of the attitude determination technique. For similar dropsonde deployments, such a reference may be readily available from an absolute density instrument, such as a sweeping Langmuir probe, carried by the nearby main payload. Notably, the attitude determination itself does not depend on this external density reference. The direction of the dropsonde angular momentum vector is constrained using the magnetometer measurements and the attitude-dependent modulation observed by the PIP, while the external density reference is introduced only during the subsequent scaling and correction of the relative PIP measurements.

The use of the measured PIP current as an attitude constraint does impose limitations on the plasma conditions under which this technique can be applied. The process of constraining the angular momentum vector relies on isolating the periodic current modulation produced by dropsonde precession from variations caused by the surrounding plasma environment. In the SpEED Demon analysis, this fit was performed in the relatively smooth density region above the sporadic E layers, where short-timescale variations in PIP current were clearly dominated by the dropsonde coning motion. Long-timescale changes in plasma density, spacecraft charging, and payload velocity were mitigated through a detrending operation prior to fitting. In regions of highly structured plasma density on temporal or spatial scales comparable to the dropsonde precession period, separating the attitude-dependent component of the signal would be substantially more difficult and could reduce the accuracy of the recovered solution.

This treatment also assumes that the GPS-derived payload velocity provides an adequate approximation of the relative ion ram direction. Significant ion velocity would alter the magnitude and direction of the flow incident on the PIP and could influence the inferred angle between the dropsonde and ion ram direction. No independent measurements of the local ion drift or neutral wind were available from the SpEED Demon dropsondes, so this contribution cannot be directly quantified for the present analysis. In the E region, ion motions are often coupled to the local neutral wind, an effect that decreases with altitude. A survey of chemical-release wind measurements by Larsen et al. [23] has shown that neutral winds in the fit regime of this analysis (i.e., >130 km) are generally less than 100 m/s. Since the payload velocities were on the order of 1 km/s, ion flow is expected to represent a comparatively small perturbation to the ram vector defined by the payload motion. In a worst-case scenario where ion flow is near 100 m/s and always perpendicular to the dropsonde trajectory, this could result in a systematic angular offset to the attitude solution of arround 5°. This value decreases for varying wind directions and more-probable wind speeds. Nevertheless, future implementation of this technique would benefit from independent measurements of ion and neutral velocities in the vicinity of the dropsopndes, if available.

This method is useful for missions in which the initial attitude of an ejectable subpayload is unavailable or unreliable. When a sufficiently accurate deployment attitude is known and can be propagated using conventional attitude sensors, a more traditional attitude determination approach may be preferable. Although the present implementation uses a Langmuir probe, the underlying approach is not inherently specific to this instrument. In principle, a similar method could be applied to other measurements having a sufficiently understood dependence on payload orientation. Such an instrument could provide an additional attitude constraint when conventional attitude information is incomplete, particularly for low-cost or low-SWaP ejectable payloads where the available attitude sensor suite is limited.

Finally, the experimental validation presented in this work is based on four dropsondes from a single sounding rocket flight and on one relatively narrow altitude interval in which the dropsondes and main payload provided approximately coincident density measurements. The results therefore demonstrate the feasibility of the technique and its particular use in the context of the SpEED Demon mission, but do not constitute a comprehensive validation across different payload geometries, plasma environments, or flight conditions. Applications to future missions, particularly those providing independent attitude, ion-flow, or neutral-wind measurements, would allow the accuracy and applicability of the method to be characterized more broadly.

## 6. Conclusions

We have presented a method of attitude determination and ion density measurement correction for small, spin-stabilized sounding rocket dropsondes using a combination of magnetometer data and Langmuir probe current measurements. The primary advantage of this technique is its lack of dependence on an initial knowledge of the spacecraft’s attitude during deployment. Nonlinear regression and optimization algorithms were applied to constrain the angular momentum vector in a manner that is physically consistent with measured data, and a model of the system was used to derive the attitude as a function of time. Data from the SpEED Demon sounding rocket campaign were analyzed and the resulting attitude solutions were validated by comparing processed density measurements between four independent dropsondes and the main payload, which does not experience significant attitude-dependent current modulations.

In this work, we did not explicitly resolve the dropsonde orientation about its long axis as the measurements of our axially symmetric Langmuir probes do not depend on the angle of this rotation. Roberts et al. [17] have previously achieved a determination of this angle using magnetometer measurements, so we neglect to repeat it here. Furthermore, in this particular application, a full attitude solution was only required for narrow measurement regions of the sounding rocket flight (in the vicinity of the sporadic E density enhancements shown in Figure 6), so these altitude regimes were prioritized in the validation process. It is likely that slight phase adjustments would be necessary to ensure a consistently accurate attitude solution throughout the entire flight. The addition of a Kalman filter [24], which combines the rotational model of the dropsonde with magnetometer and current measurements, may be useful in this regard.

Potential limitations of this technique include the presence of bulk ion velocities that could skew the ram direction as well as increased aerodynamic torques at lower altitudes. A region of smoothly varying plasma density is required to easily isolate the measurement variations caused by the dropsonde coning motion. This method of attitude determination and measurement correction is especially valuable when no knowledge of the initial payload orientation is available, and extensions of this technique could be applied to existing magnetometer-only methods to improve overall accuracy. Although the procedures discussed in this work were developed with Langmuir probe data, the approach may be generally applied to other instruments whose measurements depend on payload attitude in a similar manner.

## Figures and Tables

**Table 1:** Percent RMSE between the upleg dropsonde density profiles and the main payload density profile before and after attitude correction.

Dropsonde	Uncorrected RMSE	Corrected RMSE
1	13.5%	6.4%
2	15.1%	7.5%
3	15.8%	3.9%
4	11.4%	5.2%

## Data Availability

Dropsonde and main payload PIP data used in this manuscript are available from the SpEED Demon Mission Data Products Zenodo repository [25]. IGRF data are available from the International Association of Geomagnetism and Aeronomy [20].
